# Considerations in the search for epistasis

**DOI:** 10.1186/s13059-024-03427-z

**Published:** 2024-11-19

**Authors:** Marleen Balvert, Johnathan Cooper-Knock, Julian Stamp, Ross P. Byrne, Soufiane Mourragui, Juami van Gils, Stefania Benonisdottir, Johannes Schlüter, Kevin Kenna, Sanne Abeln, Alfredo Iacoangeli, Joséphine T. Daub, Brian L. Browning, Gizem Taş, Jiajing Hu, Yan Wang, Elham Alhathli, Calum Harvey, Luna Pianesi, Sara C. Schulte, Jorge González-Domínguez, Erik Garrisson, Lorentz workshop on epistasis, Johannes Schlüter, Ammar Al-Chalabi, Jorge Avila Cartes, Jasmijn Baaijens, Joanna von Berg, Davide Bolognini, Paola Bonizzoni, Andrea Guarracino, Mehmet Koyuturk, Magda Markowska, Raghuram Dandinasivara, Jasper van Bemmelen, Sebastian Vorbrugg, Sai Zhang, Bogdan Pasanuic, Michael P. Snyder, Alexander Schönhuth, Letitia M. F. Sng, Natalie A. Twine

**Affiliations:** 1 Tilburg University, Tilburg, The Netherlands; 2 SITraN, University of Sheffield, Sheffield, UK; 3 Brown University, Providence, USA; 4 Smurfit Institute of Genetics, Trinity College Dublin, Dublin, Ireland; 5 Hubrecht Institute, Utrecht, The Netherlands; 6 Vrije Universiteit Amsterdam, Amsterdam, The Netherlands; 7 University of Oxford, Oxford, UK; 8 Bielefeld University, Bielefeld, Germany; 9 UMC Utrecht, Utrecht, The Netherlands; 10 Department of Biostatistics and Health Informatics, King’s College London, London, UK; 11 Department of Basic and Clinical Neuroscience, King’s College London, London, UK; 12 NIHR BRC SLAM NHS Foundation Trust, London, UK; 13 University of Washington, Seattle, USA; 14 Algorithmic Bioinformatics and Center for Digital Medicine, Heinrich Heine University, Düsseldorf, Germany; 15 CITIC, University of A Coruña, A Coruña, Spain; 16 University of Tennessee, Knoxville, USA; 17 Department of Genetics, Stanford University, Stanford, USA; 18 Commonwealth Scientific and Industrial Research Organisation, Westmead, Australia; 19 University of Iceland, Reykjavik, Iceland; 20 Department of Epidemiology, University of Florida, Gainesville, FL USA; 21 Utrecht University, Utrecht, The Netherlands

## Abstract

Epistasis refers to changes in the effect on phenotype of a unit of genetic information, such as a single nucleotide polymorphism or a gene, dependent on the context of other genetic units. Such interactions are both biologically plausible and good candidates to explain observations which are not fully explained by an additive heritability model. However, the search for epistasis has so far largely failed to recover this missing heritability. We identify key challenges and propose that future works need to leverage idealized systems, known biology and even previously identified epistatic interactions, in order to guide the search for new interactions.

**Supplementary Information:**

The online version contains supplementary material available at 10.1186/s13059-024-03427-z.

## Introduction

Epistasis refers to changes in the effect of a unit of genetic information (such as a single nucleotide polymorphism or a gene) on a phenotype, dependent on the context of other genetic units. Such interactions are biologically plausible and offer a potential explanation for phenomena not fully accounted for by an additive heritability model. Heritability is a measure of the extent to which phenotypic variation is genetically determined. Broad-sense heritability refers to heritability measured by comparison of concordance rates for phenotype between monozygotic and dizygotic twins who share 100% or 50% of their genetics, respectively [1]. Missing heritability commonly refers to the gap between measured broad-sense heritability and heritability calculated by adding together the individual contributions of phenotype-associated SNPs genomewide (i.e., narrow-sense heritability). Missing heritability is important because it implies that we have an incomplete understanding of the genetic basis of health and disease. A number of possibilities could explain this missing heritability, including gene-environment interactions. Epistatic interactions are another candidate to explain a proportion of missing heritability but an alternative explanation is that current knowledge is simply missing the statistical power to discover all important additive effects. However, there is good observational evidence for epistasis, for example, from large-scale screens in yeast studying the effect of combinations of individual gene knockouts [2, 3].

A meta-analysis of twin studies concluded that for 69% of traits the data was consistent with an additive model whereby monozygotic twin correlations were almost exactly double dizygotic twin correlations [4]. However, even this study provides evidence for non-additive genetic effects in a subset of traits. For traits such as depressive disorder, hyperkinetic disorders, and atopic dermatitis, the authors observed monozygotic twin correlations which were greater than double the dizygotic twin correlations, consistent with a non-additive genetic effect. Moreover, even observations consistent with an additive model are not equivalent to actually demonstrating an additive model and the presence of an additive model does not necessarily rule out the possibility of an underlying epistatic model. Interestingly, the effect sizes of a majority of SNPs vary between genetic backgrounds [5], suggesting the presence of interactions between the genetic background and the SNP. Finally, in simulations of epistasis, additive models used to measure narrow-sense heritability fail to account for non-linear interactions between genetic variants and thus dramatically underestimate true heritability [6].

The problem is that previous searches for epistasis have so far largely failed to recover missing heritability [7]. Various computational approaches using statistics, combinatorics, and machine learning have been applied to try and detect epistasis. Each of these approaches try to address the issue of identifying relevant potential epistatic interactions from an enormous search space, either by enumerating all possibilities or by finding an efficient way to move through the search space. Consideration of epistasis inherently leads to a combinatorial explosion: the number of potential interactions increases exponentially with the number of genetic characteristics involved in each interaction.

**Fig. 1:**
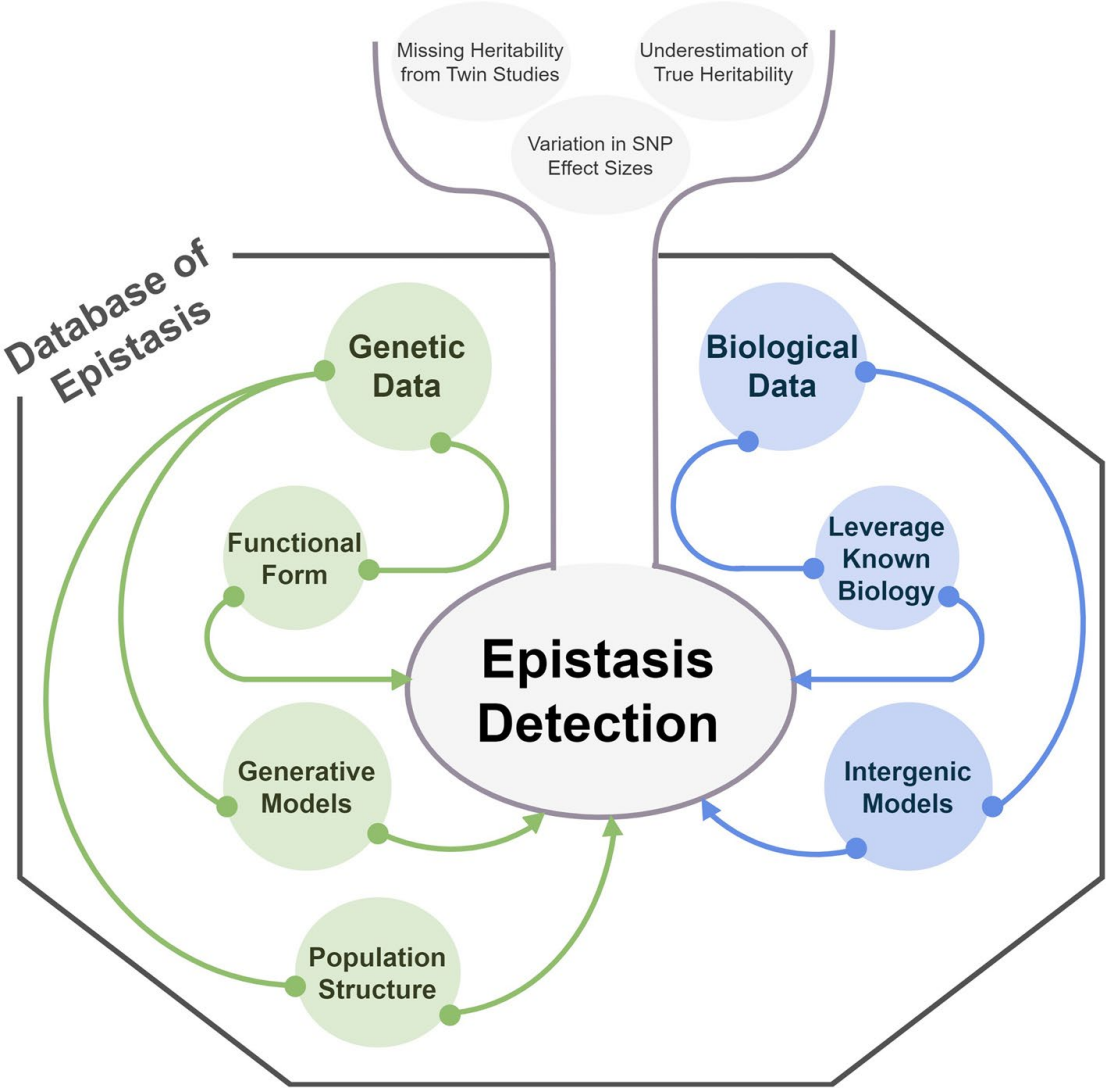
Key considerations for a comprehensive consideration of epistasis. Epistasis is posed to play a key role in genetic architecture and in the missing heritability problem. In this review, we look at epistasis from two perspectives: driven by genetic information (green circles) and by biological observations (blue circles). We discuss how genetic data can be used in a functional form or in generative models to detect epistasis, and that the inclusion of population structure information derived from genetic data is crucial. On the other hand, the discovery of epistasis can also be informed by biological observations. Ideally, both sides will lead to better detection of epistasis, ultimately leading to a key resource that is a database of epistatic interactions

During a workshop entitled “*A multidisciplinary approach to epistasis detection*,” held at the Lorentz Center in The Netherlands in July 2023, 41 experts on epistasis detection from a variety of fields came together. Through interactions and discussions, we identified challenges that need to be addressed in order to advance epistasis detection. We consider the central combinatorial challenge of epistasis identification through two perspectives: statistical and mathematical approaches to case–control studies versus leveraging biological knowledge and models (Fig. 1). Each of the two perspectives is addressed through three subtopics. For the statistical and mathematical perspective, we start by reviewing specific problems with popular model assumptions and pose the question of whether it is possible to avoid assuming any mathematical form. Next, we discuss the potential of novel generative AI models for the analysis of case–control cohort data. Third, we show empirically the importance of accounting for population structure in case–control cohort studies, which unfortunately is often overlooked. In the second half of this review, we discuss biological observations of epistasis. We start with the idea that search for epistasis should always start with biological models. Second, we discuss whether one should consider inter- and intragenic epistasis separately. Finally, we propose the use-case for a “database of epistasis” and provide guidelines for the characteristics that such a database should have.

## What assumptions of epistasis are being made and what are their implications?

Epistasis is a natural expectation of a complex system, but the search for epistasis is challenging primarily due to the combinatorial explosion of possibilities. In this section, we delve into the assumptions, mathematical or otherwise, that underpin current methods of epistasis detection and posit the use of state-of-the-art machine learning approaches in a new generation of data-driven epistasis detection methods. Many existing approaches have been recently reviewed [8]; here we extend this analysis by considering the conceptual limitations of current works and more novel approaches.

### Generalizing the functional form of epistasis

If epistasis is taken in its statistical sense as the deviation from the additive/linear baseline, then all other terms—namely quadratic and higher order interactions—are epistasis [9]. The relation between genotype and phenotype can then be represented as a function that maps a discrete sequence space onto one or more binary- or real-valued traits. Extending the formulation used in [10], a phenotype impacted by epistasis can be formulated mathematically as:

1 $$y=\sum_{a\in A}\beta_{\alpha\left(a\right)}\prod_{i\in\left(1,\cdots,N\right)}x_i^{a_i},$$

where $N$ is the total number of SNPs in the data, ${x}_{i}$ encode the SNP information (e.g., allelic dosage), *y* symbolizes the phenotype, and

$$A:=\{a\in \{\text{0,1}{\}}^{N}: {1}^{T}a\le d\}$$

with $d$ the order of the highest-order interaction. The parameter $d$ allows one to choose a maximum order for the epistatic interaction, which can be at most $N$. ${\beta }_{\alpha (a)}$ are the parameters to be estimated denoting the magnitude of the epistatic effect of the variants corresponding to the vector $a$, where $\alpha (a)$ is the index corresponding to the vector $a$ if one were to order all elements of $A$. The vector $a$ thus indicates which variants are included in the $\alpha (a)$ th interaction. For example, in the case of $N=3$ and $d=2$ this would give:

2 $$\begin{aligned} y & = {\upbeta }_{0}{x}_{1}^{0}{x}_{2}^{0}{x}_{3}^{0}+ {\upbeta }_{1}{x}_{1}^{1}{x}_{2}^{0}{x}_{3}^{0} + {\upbeta }_{2}{x}_{1}^{0}{x}_{2}^{1}{x}_{3}^{0} + {\upbeta }_{3}{x}_{1}^{0}{x}_{2}^{0}{x}_{3}^{1} + {\upbeta }_{4}{x}_{1}^{1}{x}_{2}^{1}{x}_{3}^{0}+ {\upbeta }_{5}{x}_{1}^{1}{x}_{2}^{0}{x}_{3}^{1}+ {\upbeta }_{6}{x}_{1}^{0}{x}_{2}^{1}{x}_{3}^{1}\\ &= {\upbeta }_{0}+ {\upbeta }_{1}{x}_{1}+ {\upbeta }_{2}{x}_{2}+ {\upbeta }_{3}{x}_{3}+ {\upbeta }_{4}{x}_{1}{x}_{2}+ {\upbeta }_{5}{x}_{1}{x}_{3}+ {\upbeta }_{6}{x}_{2}{x}_{3}\cdot\end{aligned}$$

Note that since $d=2$, the interaction between all three variants is not included.

In other words, epistasis is the combined effect of any combination of SNPs up to a certain order of magnitude. For binary traits, one can apply the logit function to the right-hand side of (1). Note that explicitly using formulation (1) leads to a combinatorial explosion in the number of terms and hence parameters to be estimated as the number of SNPs and the degree $d$ increase, hence explicitly estimating the effect of all interaction terms is infeasible.

Any function can be represented as a series expansion with the commonly used Taylor and Fourier series expansion [11]. The difference between the two representations for modeling epistasis is the reference frame. The Taylor series uses the wild type as reference to quantify epistatic interactions and in the Fourier series epistatic effects are averages over all backgrounds [12]. With epistasis, a wide body of literature suggests that many different mathematical formulations can be linked using the weighted Walsh-Hadamard transform [12].

Models that identify epistatic interactions from genotype data nearly always make assumptions on the form of the epistatic relationship (Table 1). There is a gradient in current approaches of epistasis detection: from models assuming a specific form of epistasis (e.g., BOOST [13], BitEpi [14], Fiuncho [15], IRELAND [16, 17], MDR [17]) to models that learn an epistatic relationship of any form (e.g., [12, 18–20]). These assumptions are described in the paragraphs below.

**Table 1:** Table summarizing the assumptions made by various methodological approaches and tools for detecting epistasis

Assumption	Approach/tool
Two-way interaction only	BOOST, GenEpi, EpiGWAS, MDR
Two-, three-, or four-way interactions	BitEpi (4-way), LOBICO (4-way), MDSN (3-way)
Any order of interaction	IRELAND, Fiuncho
Highly polygenic/omnigenic trait	Deep neural nets
SNP interaction partners are independent	Random forests; any tool which does not account for population structure
Variant interactions can be described through a combination of decision trees	Random forest
Cases can be separated from controls through a hyperplane in the (modified) variant space	Support vector machine
Epistatic interactions are described through logic statements	LOBICO, IRELAND

Approaches that assume a specific form of epistatic interaction, for example, pairwise, triplet, or quadruplet interactions, are often easier to understand and can provide directly interpretable outcomes. However, they still suffer from the combinatorial explosion if there is no constraint on the type and number of interacting variants. Consequently, several methods focus on two-way interactions only [13, 21, 22] while other more exhaustive search methods are limited by the computational complexity of the approach and often do not go beyond four-way interactions [14, 15, 23, 24]. Fiuncho and IRELAND do go beyond four-way interactions [14–17, 23, 24], though they are limited in the number of SNPs they can analyze simultaneously. Whether going beyond four-way interactions is clinically relevant and can be validated beyond statistical evidence remains an open question. It is, however, biologically plausible that many SNPs are involved in the same epistatic interaction [25, 26].

On the other hand, freeform approaches such as deep neural networks (DNNs) [19, 20, 27, 28], as supported by mathematical theorems (the universal approximation theorem, e.g., [29, 30]), can approximate arbitrary functional relationships, thereby in theory they can avoid the requirement to impose any assumption in terms of functional relationships driving epistasis. This makes them more flexible and less prone to computational limits. In practice, however, these approaches require additional steps, perhaps yet to be developed, to not only implicitly capture but also provide an explicit description of the epistatic interaction [18]. Because DNNs tend to use a large number of input variables for phenotype prediction, they arguably assume a highly polygenic or even omnigenic trait [29] in practice.

Many classical machine learning (ML) approaches sit between these two extremes, such as decision tree ensembles, i.e., random forest [31–35], boosting [36], and support vector machines [37–40]. These approaches make mild assumptions on the functional form of the epistatic interaction, allowing them to deal with higher-order interactions. For random forests, these assumptions include that SNPs forming interactions must be independent of each other. Random forests also assume that the relationship between epistasis and genetic variants can be described as a combination of decision trees, while support vector machines assume that one can separate cases from controls by using a hyperplane in the (transformed) variant space.

The standard formulations of epistasis presented above link genotype to phenotype by means of statistical models, machine learning approaches, or combinatorics, all based on large datasets. However, the joint probability structure of the dataset is never explicitly exploited during inference. Modelization and subsequent dissection of the joint probability between genotypic features, and between genotypic features and a phenotype of interest, offer another approach to study epistasis.

### Using generative approaches to model and explore epistasis

Apart from the classical ML approaches aforementioned, recent algorithmic and computational advances have offered insight into the potential of deep generative models in genetics [41, 42]. Inspired by many successful applications in other scientific fields [43–45], we envision that leveraging generative approaches could offer a transformative approach to identify genetic interactions. Note that, just as for DNN based classifiers, universal approximation theorems for probability distributions [46] support the utmost flexibility of generative deep learning approaches. A large part of the advantage of this approach rests on the ability to perturb a latent space representation of genetic interactions and make observations regarding the effect on phenotypes: in effect providing an experimental system with a tractable number of variables. We explain and explore this idea below.

**Fig. 2:**
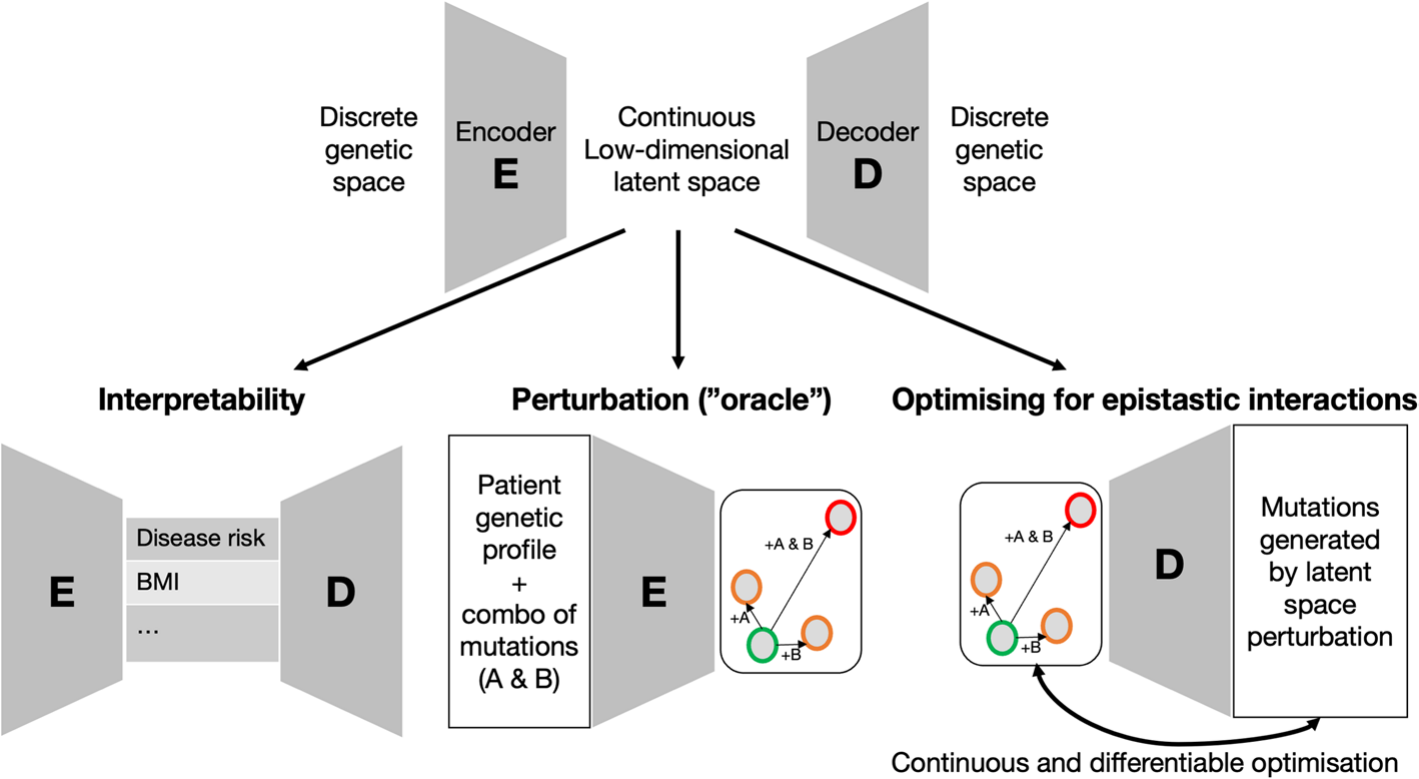
Example of how generative modeling can be employed to hunt for genetic interactions. Most deep generative models are made of two elements: the encoder, which reduces dimensionality, and the decoder, which can generate genetic profiles in silico (top panel). We present three problems where generative models can be employed. Interpretability: The output of the encoder, and input of the decoder, can be interpreted and related to phenotypes of interest. Perturbation: A patient’s genetic profile can be perturbed in silico and passed through the encoder. For instance, a patient with two wild-type alleles (green circles) can be modified by induction of A or B (orange circles) or both at the same time (red circles). Study of the corresponding perturbation in the latent space can help prioritize potentially interacting genetic pairs. Optimization: Finally, a deep generative model could be directly employed inside an optimization strategy geared towards finding epistatic interactions, benefiting from two advantages of deep generative models: the auto-differentiation of the decoder and the continuous character of the latent space

A deep generative model aims to construct a condensed representation of the genetic information that accurately describes the distribution of genetic variance in the population from which observed genetic data is sampled. That is, the generative model learns how to represent an individual’s genetic information in a condensed manner with minimal loss of information, meaning that it can reconstruct the original genetic information from this condensed representation with high accuracy. Deep generative models are typically composed of three components: the encoder, the latent space, and the decoder (Fig. 2). Firstly, the encoder maps a sample to a space of much lower dimensionality. This so-called latent space offers an intriguing property—it is continuous, unlike the binary nature of genetic profiles (wild-type or mutated). Finally, the decoder takes a point within this latent space, whether it originates from the encoder or is chosen randomly, and reconstructs the corresponding sample. Note that this sample could be non-existent if the point in the latent space is chosen randomly. Although deep generative models come in various styles [47–49], the roles of the encoder and decoder may differ, but they all share these fundamental characteristics.

We foresee three kinds of applications that require the development of models with a disentangled latent space [48, 50]. Firstly, extrapolating from protein structure work, we expect that well-designed models will exhibit emergent information in the latent space [51]. In more concrete detail, using interpretable labels (such as diseased/non-diseased), one trains the encoder to map differently labeled data to distant parts of the latent space, while placing identically labeled data near to each other. Such training procedures can be implemented by the integration of contrastive loss functions [52] into the training of the encoder-decoder architecture. As per their definition, contrastive loss functions are exactly the drivers that keep similar things together, while keeping separate things apart when embedding data into latent space. As a result, the interpretability of the model obtained by an appropriately structured continuously valued latent space could be instrumental in increasing the power of standard analysis (Fig. 2, Interpretability). For example, linking parts of the latent space to known phenotypes (e.g. disease risk) could aid in identifying new disease-risk regions. This expansion of the available dataset would enhance the power of standard epistasis detection analyses. A second, more direct application of such a model involves using it as an “oracle” that provides quantitative insights into the perturbation caused by a pair of genetic alterations (Fig. 2, Perturbation). For instance, given two alleles A and B at different loci, one could measure the perturbation in the latent space induced by each mutation and compare it to the perturbation in the latent space caused by A and B combined. If the combined mutation leads to the same perturbation as the two individual combinations together, then there is no indication of epistasis, else there is. Lastly, the model can be used in a more exploratory manner through the design of optimization routines (Fig. 2, Optimization). Using the decoder’s gradients enables the identification of genetic pairs that lead to maximal perturbations in the latent space, indicating interaction within these pairs and hence identifying potential epistatic interactions. These three directions, far from being exhaustive, showcase the potential of deep generative models in the detection of epistatic interactions. Since the use of these methods in genomic applications is still in its infancy but highly promising, extensive further research along these lines is necessary.

### Population structure confounds regression-based epistasis detection

In addition to the assumptions on the form of the epistatic interaction, there are underlying assumptions of genetic data that should inform epistasis detection models, particularly linkage disequilibrium (LD). However, many epistasis detection datasets and tools fail to account for LD structures which means they will be particularly vulnerable to population mismatch. Here we include a detailed consideration of this failure within epistasis detection and how this could be addressed.

**Fig. 3:**
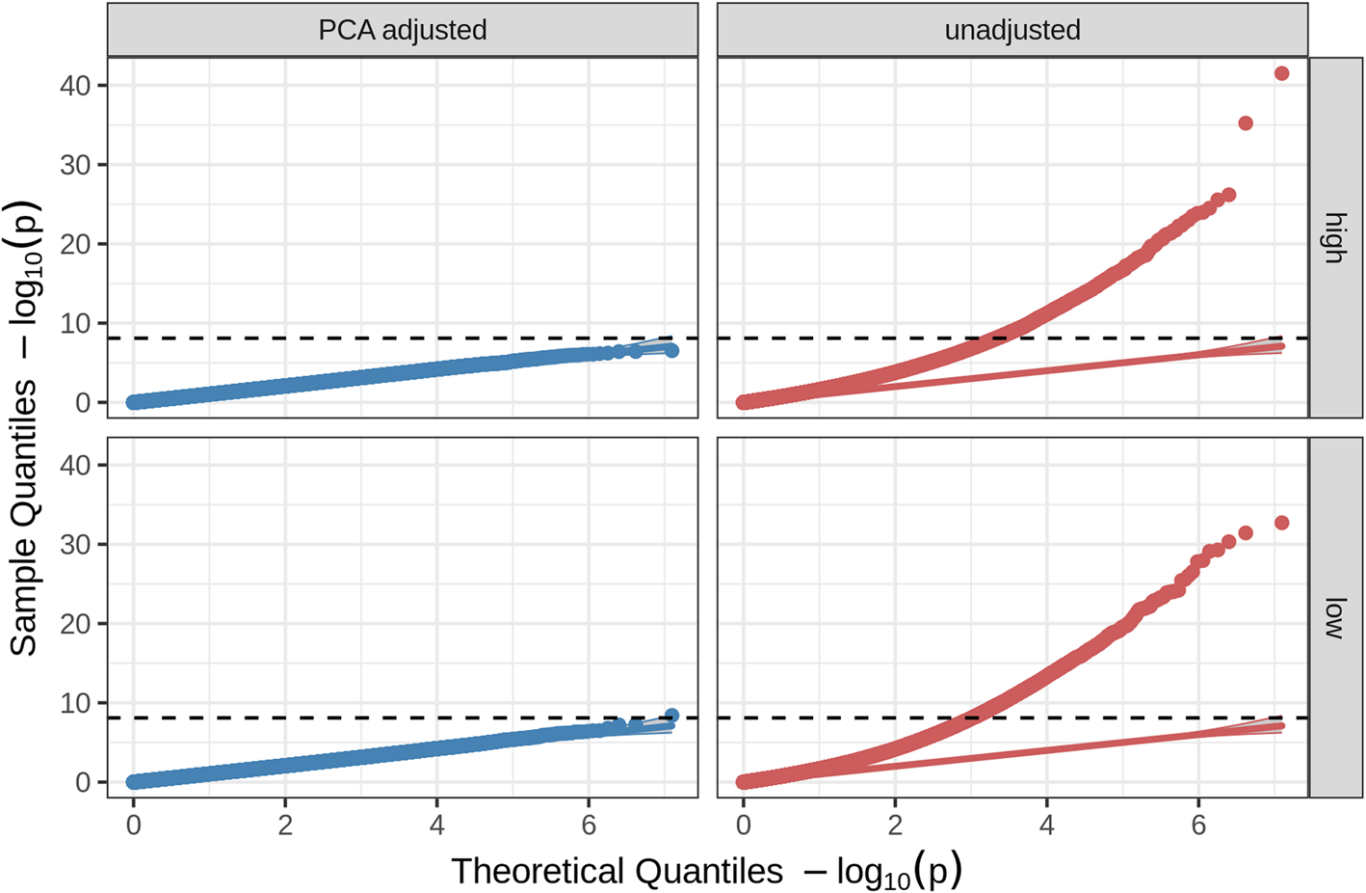
Population structure confounds regression-based epistasis detection. QQ plots for PLINK pairwise epistasis analysis on simulated null data with population structure and no true epistatic effects (interactions), i.e., only additive contributions (main effects), and trait heritability of 0.5 (details of simulation in Additional file 1: Supplementary Methods). Comparison of analysis corrected for population structure with 20 PCs (blue) and uncorrected for population structure (red) shows that population structure also leads to inflation of small *p* values and a large number of false positives in regression-based genome-wide association studies that model epistasis as a pairwise interaction term. The dashed horizontal line is the significance threshold after Bonferroni correction. Phenotype adjustment here is performed by regressing the phenotype against 20 PCs in a multiple linear regression model and using the residuals as the “adjusted phenotype.” The facets labeled as “high” and “low” correspond to 1000 and 100 true causal variants respectively with additive-only contributions to trait variance

Events in human evolutionary history such as migration and admixture [53] are reflected in differences in allele frequencies (AFs) between different populations [54]. The concept of genetic populations is a simplified description of these genetic patterns [55]. The differences in AFs between populations are called population structure and have been described as a confounding factor in genome-wide association studies (GWAS) [56–59]. In a naive association test, the samples are modeled as independent, an assumption that cannot hold when there are such systematic genetic trends within the data. Epistasis analysis, similar to GWAS, is vulnerable to confounding from population structure, which if uncorrected, can result in substantial *p* value inflation and false positives in analyses with no true epistatic interactions (Fig. 3). Furthermore, previous research has shown that a slight change in the AF of a SNP results in a substantial decrease in power to replicate the main effects of said SNP when there is an underlying epistatic model [60]. Detection and correction of population structure are thus of core importance to the study of epistasis. We propose that solutions to this problem can be informed by common practices from GWAS analysis.

In GWAS, there are two main approaches to correcting for population structure. The first includes principal components of genetic similarity as additional covariates in a linear model. Principal component analysis (PCA) aims to explain the variance–covariance structure of a high-dimensional data set with a relatively small number of linear combinations of the original variables [61]. The first few principal components of genetic data often capture population structure and are suitable covariates for correcting this source of confounding [62]. The second includes a random effect that is informed by the genetic covariance between samples in a linear mixed model (LMM) approach. By including a random effect that covaries with the genetic similarity, the samples are no longer modeled as independent. This method, while computationally more costly, is able to account for population structure without overfitting; in the presence of cryptic relatedness LMMs outperform principal component-based correction methods [57, 63].

Analogous methods to those used in GWAS have been adopted to account for population structure in some epistasis detection approaches, including methods adopting PCA correction (e.g., MBMDR-PC [64]) and LMMs (e.g., REMMA-epi [65] and FaST-LMM-epi [66, 67]). Indeed, LMM approaches have been shown to produce significantly lower statistical inflation than PLINK’s pairwise epistasis method [65, 68]. Surprisingly, several commonly used methods for epistasis detection including PLINK epistasis and BOOST [13] do not have a built-in option for including covariates or otherwise correcting for population structure.

Our simulation analysis suggests that simply ignoring population structure in these cases is unwise and would lead to substantial statistical inflation and false positives (Fig. 3, Additional file 1: Supplementary Methods). Here we simulated traits with no true epistatic effects (only additive effects) in a structured population and performed plink epistasis detection to evaluate the impact of population structure on the resulting test statistics. We expect that if epistasis tests were inherently robust to confounding from population structure there would be no significant hits or *p* value inflation, as no epistasis was simulated. For population structure correction, the phenotype was adjusted using multiple linear regression on the first 20 PCs prior to analysis. The simulation code is open source at https://github.com/jdstamp/leiden_paper. QQ plots of our simulations show evidence of statistical inflation and large numbers of false positives only in the simulations with no correction for population structure (Fig. 3, “unadjusted” panels on right), indicating that population structure can confound epistasis detection methods, while analyses corrected for structure were well-controlled (Fig. 3, “PCA adjusted” panels on left). We thus recommend that researchers using methods without built-in population structure correction for epistasis analysis address population structure, for example, by first adjusting their phenotype using principal components (taking the residuals from a multiple regression), or alternatively using a suitable LMM approach.

## Leveraging biology in the search for epistasis

The first part of this review focused on using statistical and mathematical approaches to identify epistasis from data. In this second part, we focus on if and how biological information can be leveraged to look for epistasis. We pose two questions surrounding the use of model systems and intergenic versus intragenic mechanisms in the search for epistasis, followed by a discussion on the usefulness of a database of epistasis.

### Should a search for epistasis start with biological observations?

We assert that conclusive evidence for the role of epistasis in determining disease heritability has come from interactions that have been identified in large case–control cohorts and model systems such as cell lines and organisms. We hypothesize that true epistatic interactions will be observable in both model systems and case–control cohorts. However, false positive epistatic interactions may be more likely in case–control datasets where, for example, population structure is imperfectly matched. On the other hand, the potential challenge with model systems is knowing whether the readout and the cell/tissue context are a correct approximation of disease. Indeed, a model system may suggest an epistatic interaction which is specific to the genetic background of the model organism and may not be important in an outbred population.

Many double mutant genetic knockout screens have been performed, both in model organisms and human cell lines [69]. The problem with the use of organisms for epistasis detection is, again, the size of the search space. Extensive large-scale screens have been performed in yeast, focusing on large-scale characterization of cells with combinations of two or more individual gene knockouts or temperature alleles [2, 70, 71]. Such screens have proved useful, for example, in delineating biological pathways containing genes with similar interaction profiles. However, they have not, as yet, provided the scale necessary for an exhaustive search for epistasis. Indeed higher organisms such as mice are not at all suitable for large-scale screens due to the practical undertaking involved in exhaustive characterization.

Unlike organisms, cells are more tractable. In mammalian cells, many double mutant screens have been performed, mostly in the context of cancer where gene knockouts have important therapeutic implications since gene knockouts can represent drug-targeting conditions. For example, in one recent study double mutant screens were performed for ~ 34,937 gene pairs in MCF-10A breast cell lines, and their effects on tumor growth were examined in mice [72]. Statistically significant gene pairs were identified and grouped into interacting modules. Interestingly, the genes within a group exhibited epistatic effects on gene expression of other group members. Overall, this study revealed the gene interaction network of tumor growth and has important implications for therapeutic strategies. Another recent work examined 1191 putative functional gene pairs and/or paralogs in human melanoma lines and identified 109 pairs that affected fitness [73]. An important consideration in a biological experiment is context, for example, epistatic interactions may only be apparent in a specific environment; when that environment is the presence of a particular toxin or therapeutic, this observation can be used to identify epistatic interactions which have the potential to guide personalized medicine [74].

An important and well understood biological consideration is the separation between intergenic and intragenic mechanisms of epistasis. There is good evidence for intragenic interactions such as haplotypes associating with altered gene expression depleted for deleterious coding alleles [75]. Similarly, there is evidence for intergenic epistasis, particularly between genes of similar function [76], where an established example concerns mutations within different hemoglobin beta-chains [77]. Intergenic epistasis across different functional pathways also tends to be the result of compensatory adaptation [78] and typically genes within the same pathway have a similar interaction profile [70]. The problem with separating intergenic and intragenic epistasis is that both are combined in real-world biological systems. To circumvent this, we suggest a stepwise approach where intergenic epistasis is analyzed and identified before searching for intragenic epistasis within each intergenic interaction.

An alternative framing is that the search for epistasis should prioritise variants where biological evidence for an effect is provided by a case-control cohort instead of a model system. If there is an epistatic interaction, we might expect to be able to measure the association between phenotype and genotype even with only one of the involved variants. Intuitively, this will depend on the frequency of the alleles in question, the size of the study population, and the effect size of the genetic variants. If true, then we should be able to use prioritization methods based on independent models (i.e. an additive model) to reduce the search space size for epistasis. One study has already applied this principle [21] where the search for epistasis was focused on additional genetic variants which increased the effect of another, significant in isolation, genetic variant. Their results are promising, showing that in several datasets this method outperforms GBOOST and Lasso. A future extension for this approach might be to use symbolic regression [79, 80] to detect epistasis between genetic variants with nominal significance and to determine the mathematical formulation of the relationship without a pre-specification.

### A database of epistasis

An obvious approach to leveraging known information in the search for epistasis is to use the literature of known epistatic interactions. There have been efforts to collect large amounts of epistasis data in one platform. For example, using pre-selected gene-specific transcription factors in *Saccharomyces cerevisiae* [3], SynLetDB provides a database specifically for synthetic lethality cases. However, most studies report genome-wide epistasis which are restricted to one organism or are phenotype-specific such as Alzheimer’s disease [81, 82] or in cancer [83], and are not collated and standardized into a single database. A database that does exist for epistasis across multiple diseases is driven by a single methodological approach (https://epistasis-disease-atlas.com, [84]), therefore is limited in the types of epistatic interactions it contains. Furthermore, it remains difficult for researchers to reuse epistasis data because of the different definitions of epistasis, and different experimental and computational techniques used to identify epistasis.

**Fig. 4:**
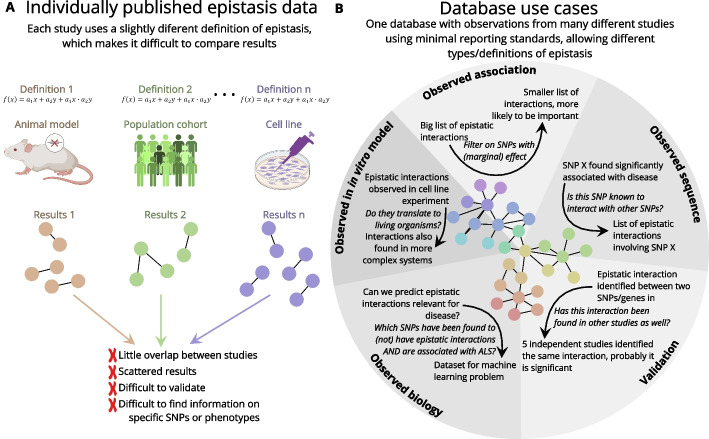
The need for an epistasis database. **A** It is currently difficult to compare epistasis data between studies, as there are many different approaches, models, and even definitions of epistasis. **B** Use cases of an epistasis database. By collecting epistasis data into one coherent framework, researchers can more easily find relevant information about their genes/SNPs and interactions of interest. Additionally, collecting epistasis data into one large framework would benefit from the creation of a reporting standard for epistasis data, such that epistasis data can be more easily collected and reused in the future

We argue that although studies on epistasis can be highly diverse, there is a core set of data and metadata that can and should be reported for all studies to be able to effectively leverage known epistatic interactions. This set of minimum reporting standards could be based on other guidelines available such as MIAME/MINSEQE [85] that are used in sequence-based platforms, for example, the EGA (https://web2.ega-archive.org/) or GEO (www.ncbi.nlm.nih.gov/geo/), and should include metadata per study, per sample, and per interaction, as outlined in Table 2. If all studies publishing epistasis information adhere to the same set of minimal reporting standards, referencing and using known epistasis would become much simpler. Additionally, it would facilitate the collection of epistasis information into one large database, which would benefit many researchers in the field of epistasis. Because epistasis is such a complex phenomenon, generating a database would be helpful to explore available data or validate new results (Fig. 4B). Additionally, a database could identify genes of interest, or other studies using a specific method of epistasis detection.

**Table 2:** The set of minimum reporting standards for a database of epistasis

Per study	[1] Phenotype description [2] Experimental method [3] Computational method [4] Study sample size
Per sample	[1] Organism/cell line [2] Sample description
Per interaction	[1] SNP ID or SV gene loci [2] Associated HUGO gene symbol [3] Value of phenotype measure (e.g., gene expression, disease, trait, cell growth) [4] Confidence or significance score [5] Validation status [6] Nature of interaction (e.g., pairwise/higher order)

It is important to consider which types of researchers may use a database of epistasis. For example, researchers studying a certain gene or SNP may want to query that gene/SNP to get an overview of potential epistatic interactions, while studies identifying epistatic interactions may query specific interactions to validate their findings, or search for orthogonal sources of validation, e.g., knock-out studies for specific interactions demonstrating a phenotypic effect or reduced expression level at the mRNA or protein level. On the other hand, researchers focusing on a specific disease or other phenotype may query that phenotype to find any associated interactions.

However, because epistasis is such a diverse phenomenon, creating a comprehensive database to capture this information poses several challenges. For example, it would be ideal for a database to contain curated positive (i.e., epistatic interaction occurs) and negative (i.e., epistatic interaction does not occur) cases. A further complication is that computational methods use a range of measures of confidence for epistatic interactions (e.g., statistical significance (*p* value/FDR), feature weight, knock-out experiments), and thus defining and standardizing positive and negative epistatic interaction is not straightforward. Likewise, these computational methods use different approaches (e.g., neural nets, random forests, regression), and thus have different metrics to detect epistasis, which may not be directly comparable (Fig. 4A). Hence, the provenance of each epistatic interaction, as suggested in Table 2, is essential to filter data and find epistatic interactions with multiple sources of evidence.

## Conclusion

The search for epistatic interactions which influence disease heritability is challenging but essential to facilitate effective personalized medicine. We have outlined some of the challenges, particularly the intractability of modeling epistasis. While we have suggested some modeling approaches that may be fruitful in the future, we have also considered real steps that could be used to improve models by integrating known biology. In particular, we suggest that all models of epistasis in the future should include an explicit correction for population structure and we show the importance of this through simulation. We make the case for a database of epistasis to bring together what is already known in the hope that this is a firmer foundation for discovery than strict model definitions, which may or may not be representative. Using generative models may be a way in which these biological observations can be summarized in a meaningful fashion.

## Supplementary Information

Additional file 1. Supplementary methods for simulation of the effect of population structure on detection of epistasis, based on models in [88] and [89].

Additional file 2. Review history.

## Data Availability

The code for the simulated data is available at Github [86] and Zenodo [87] under an MIT license.
