# Author Correction: Considerations in the search for epistasis

**DOI:** 10.1186/s13059-025-03477-x

**Published:** 2025-01-20

**Authors:** Marleen Balvert, Johnathan Cooper-Knock, Julian Stamp, Ross P. Byrne, Soufane Mourragui, Juami van Gils, Stefania Benonisdottir, Johannes Schlüter, Kevin Kenna, Sanne Abeln, Alfredo Iacoangeli, Joséphine T. Daub, Brian L. Browning, Gizem Taş, Jiajing Hu, Yan Wang, Elham Alhathli, Calum Harvey, Luna Pianesi, Sara C. Schulte, Jorge González-Domínguez, Erik Garrisson, Lorentz workshop on epistasis, Michael P. Snyder, Alexander Schönhuth, Letitia M. F. Sng, Natalie A. Twine

**Affiliations:** 1 Tilburg University, Tilburg, The Netherlands; 2 SITraN, University of Sheffield, Sheffield, UK; 3 Brown University, Providence, USA; 4 Smurfit Institute of Genetics, Trinity College Dublin, Dublin, Ireland; 5 Hubrecht Institute, Utrecht, The Netherlands; 6 Vrije Universiteit Amsterdam, Amsterdam, The Netherlands; 7 University of Oxford, Oxford, UK; 8 Bielefeld University, Bielefeld, Germany; 9 UMC Utrecht, Utrecht, The Netherlands; 10 Department of Biostatistics and Health Informatics, King’s College London, London, UK; 11 Department of Basic and Clinical Neuroscience, King’s College London, London, UK; 12 NIHR BRC SLAM NHS Foundation Trust, London, UK; 13 University of Washington, Seattle, USA; 14 Algorithmic Bioinformatics and Center for Digital Medicine, Heinrich Heine University, Düsseldorf, Germany; 15 CITIC, University of A Coruña, A Coruña, Spain; 16 University of Tennessee, Knoxville, USA; 17 Department of Genetics, Stanford University, Stanford, USA; 18 Commonwealth Scientific and Industrial Research Organisation, Westmead, Australia; 19 University of Iceland, Reykjavik, Iceland; 20 Department of Epidemiology, University of Florida, Gainesville, FL USA; 21 Utrecht University, Utrecht, The Netherlands

**Correction: Genome Biol 25, 296 (2024)**

**https://doi.org/10.1186/s13059-024-03427-z**

Following publication of the original article [1], the authors identified that two author affiliations were incorrect.

Joséphine Daub is affiliated with Utrecht University (21) and not affiliation 9.

Sanne Abeln is affiliated with Utrecht University only (21) and not affiliation 1.

The original article [1] has been corrected.
