# Supplementary material for: Considerations in the search for epistasis
Source: Genome Biol. 2024 Nov 19;25:296. doi: 10.1186/s13059-024-03427-z (PMC11574992; doi:10.1186/s13059-024-03427-z)
Supplement: Supplementary file 1 — Additional file 1. Supplementary methods for simulation of the effect of population structure on detection of epistasis, based on models in [88] and [89]. [file 13059_2024_3427_MOESM1_ESM.docx]

**Supplementary Methods** for simulation of the effect of population structure on detection of epistasis

To examine the importance of adjusting for population structure in epistasis analysis we simulated genotype and trait data with no epistatic effects and tested for epistasis in scenarios with and without correction for population structure as follows. Genotype data were simulated for 5000 individuals split evenly across 4 populations using the python packages msprime (<https://github.com/tskit-dev/msprime>) and stdpopsim (<https://github.com/popsim-consortium/stdpopsim>) under the demographic model for American admixture [[88]](https://www.zotero.org/google-docs/?vK1O8h), which extends a model of African, European, Asian demographic history [[89]](https://www.zotero.org/google-docs/?ktr8LS). Traits with additive components (main effects), but no epistatic components (interaction effects) were simulated using the software GCTA v1.94.1 ( <https://yanglab.westlake.edu.cn/software/gcta/#Overview>), with a fixed trait heritability of 0.5 and either 100 or 1000 causal variants. Epistasis detection GWAS was performed for each simulated trait replicate (10 replicates per parameter set) using PLINK v1.90 (<https://www.cog-genomics.org/plink/> ; --epistasis flag) both with and without correction for population structure. For the analyses corrected for population structure (**Figure 3**; “PCA adjusted”), the phenotype was first adjusted using multiple linear regression on the first 20 PCs prior to epistasis analysis. QQ-plots were used to assess the statistical inflation of the adjusted and unadjusted scenarios. The simulation code with all relevant parameters is open source at <https://github.com/jdstamp/leiden_paper>.
