# Supplementary material for: Considerations in the search for epistasis
Source: Genome Biol. 2024 Nov 19;25:296. doi: 10.1186/s13059-024-03427-z (PMC11574992; doi:10.1186/s13059-024-03427-z)
Supplement: Supplementary file 2 — Additional file 2. Review history. [file 13059_2024_3427_MOESM2_ESM.docx]

**Round 1**

**Reviewer 1**

The review is logically written and offers valuable synthetic information and insights for future research directions. It would benefit readers interested in genotype-phenotype mapping, particularly with the interest in epistasis.

Major Concerns:
1. Vague Explanation of Assumptions: The manuscript fails to explicitly state the assumptions underlying different methods for detecting epistasis, despite the title being 'Are we making the right assumptions about epistasis?'.
Throughout the manuscript, assumptions are vaguely mentioned as 'assume a specific form' or 'some assumption,' but the exact nature of these assumptions is not specified. This lack of detail makes it difficult for readers to evaluate whether these assumptions are correct. The only clear exception is the discussion on population imbalance, where some methods assume a balanced population, which is well explained.

2. Scope of Epistasis: The manuscript focuses on genetic interactions in disease heritability, as indicated in Line 57 and Section 2. The hypothesis that "true epistatic interactions will be observable in both model systems and case-control cohorts" (Line 59, Section 2) is only valid within this context. This is because epistasis can vary in different environments and cell types (as cited in ref 77). However, the specific scope of the review is not clearly stated in the title, abstract, or introduction, leading to potential confusion for readers.

Some specific points need revision:
1. Introduction:

The authors discuss epistasis in the context of missing heritability. Since many other factors, besides epistasis, contribute to missing heritability, the authors should also mention genotype-environment interactions to avoid misleading readers who are not experts in genotype-phenotype relationships and heritability.

2. Section 1.1 Formula:

The formula presented is problematic. It is described as 'epistasis formulated mathematically,' implying that y represents epistasis. However, y typically symbolizes the quantitative phenotype, and 'e' (or any other symbol) represents epistasis. The term βα(a) is defined as parameters to be estimated, but their biological significance is not explained. It appears that βα(a) signifies epistasis; if so, this needs clarification.
Intrinsic Relationship Between N and d: The manuscript does not address the theoretical relationship between N(number of SNPs) and d (the highest order interaction). For example, theoretically, the highest-order interaction could be d =N−1.

If the authors mean that one can set a maximum highest-order interaction to evaluate (which should be smaller or equal to d=N-1), this should be explicitly stated.
Formula (1): In lines 57-60, shouldn't the term β7(x1)^1(x2)^1(x3)^1 also be considered? Otherwise, it appears that only the first-order interaction terms (between two SNPs) are included, without accounting for three-way interactions (involving all three SNPs together).

3. The manuscript would benefit from additional tables or boxes. Currently, it is unclear what specific assumptions are made by each method of detecting epistasis.

Throughout the manuscript, particularly in the first part, different methods of detecting epistasis are described with phrases like 'assume a specific form' or 'milder assumption', but the exact nature of these forms and assumptions is not specified.

Including a table or box summarising this information would provide clarity.

4. Some minor points:

The first equation (line 41) should be indexed so that the current equation (1) becomes equation (2).
It is unclear whether the colors of the circles in the lower panels have any significance. If they do, this should be explained.

**Reviewer 2**

In "Are we making the right assumptions about epistasis?", Balvert, Cooper-Knock and colleagues describe a couple of key challenges to detecting epistatic interactions in genomic data. They go on to propose methodological approaches and research infrastructure that would help to identify epistatic interactions in future research. As stated in the introduction, this article summarizes key discussions and conclusions from a recent workshop on this topic, so it will likely be of interest to researchers in the field focusing on the role of epistasis in phenotypic and disease variation in humans.

The authors primarily focus on the role of epistasis in disease heritability estimation and interpretation. While this is certainly an important topic in human medical genetics, it may be worth pointing out that epistasis plays an important role in broader evolutionary theory as well.There are many examples of the role of epistasis in evolutionary biology, both theoretical and empirical, that could be highlighted and potentially broaden the appeal of the review. Many of the challenges for detecting epistatic interactions in non-human-disease settings are the same as those discussed here.

This review article is in large parts well-written, though it could probably use a careful reading for language and clarity. Most sections have a good balance of the "big picture" challenges and suggested approaches to tackling those challenges, and often provide helpful examples or spell out some of the details. However, I believe a few of the sections could be significantly improved (listed below).

Section 2.2: While it is pointed out that both intergenic and intragenic epistatic interactions have been documented, the title of the section, "Should we focus on intergenic vs intragenic interaction separately?", (despite being a bit confusingly worded) isn't actually answered. Or rather, the proposed solution is given in a single sentence without justification or detailed expansion. I was left wondering what the point of this section is.

Section 2.3: A database of epistasis. The authors make an argument for such a database and suggest guidelines and approaches for making it useful to researchers, in addition to challenges. Is such a database in the works? If so, and especially if it is a product of or was first envisioned in the course of this workshop, that could be discussed here.

Title: The title doesn't seem to be all that connected to the majority of the content of the review.

Figure 1: I don't find this figure particularly intuitive or informative. I do think that design such conceptual figures is very challenging, but as presented, I don't see it as providing any information beyond a "word cloud". It was difficult to understand the lines connecting the different elements, and the legend didn't clarify things for me (although, such a figure is ideally understandable even without reading the legend).

I struggled to wrap my head around the interpretability aspect of the latent space from the encoder. It could be helpful to make this a more concrete, either with an example or hypothetical, for those of us less familiar with interpreting machine learning or generative models.

**Reviewer 3**

Balvert et al present a well written overview of many issues regarding the reality of and the detection of epistasis. The likelihood that epistasis is an important factor in phenotypic variation is a long standing issue especially among human geneticists and as such makes this contribution overall worthy. Below are several comments I think should be more explicitly dealt with in the paper that may clarify some issues.

The first point is that in the abstract it is stated that the role of epistasis in phenotypic variation is controversial. Although true to some extent, this is predominantly true among human geneticists. Research on non-human systems is generally accepting of epistasis.
Another point both in the abstract and introduction is the role of epistasis in the missing heritability. Most arguments about missing heritability have focused on narrow sense heritability, which of course does not explicitly include epistasis. The authors should clarify the role of epistasis in heritability - both, broad where it is explicitly considered and narrow where it may be included, but only implicitly. Specifically, detecting a significant additive model is not necessarily inconsistent with an underlying true epistatic genetic model. The question relates to what is being addressed, understanding genotype-phenotype maps or simply trait prediction.

In the discussion of epistasis in yeast a very old citation is used. This is fine but more recent ones, such as, should also be included. For example, Kuzmin et al Science 2018 and Costanzo et al Science 2016 - there are several others from this group alone. This other work also brings up the point of merging detection of epistasis with explicit pathway analyses - not discussed in the paper. This is however key to using biological knowledge to understand epistasis and whether it is important.

The issue of population structure and epistasis detection has also been addressed in prior literature in terms of how varying allele frequency of and interacting par affects detection likelihood of detecting marginal effects in spite of an underlying interactive model. For example, CS Greene et al PLOS One 2009 perform simulations to show that background is important for both detection of marginal effects but also interaction. This goes to the point above - does detecting additive effects nullify the likelihood of an underlying interaction. In some ways this is the core of the problem.
Lastly, the authors should note in the discussion of animal models and cell lines that these are interesting but not necessarily reflective of nature. In these experiments the backgrounds are usually fixed and in general when examined interactions will be found if there are any kind of biological interactions. However, in outbreeding samples such as case-control studies even if present the same biological interactions may be missed by the resilience of the genetic structure of the population. Similarly, even if two molecular interact, they can only be detected if both are variable in a given sample.

Overall, this is an excellent overview but not comprehensive. Although being completely comprehensive is not possible in such a short piece, several ideas as noted above should probably be addressed to improve the completeness of the arguments.

**Authors’ response**

Reviewer #1:

The review is logically written and offers valuable synthetic information and insights for future research directions. It would benefit readers interested in genotype-phenotype mapping, particularly with the interest in epistasis.

Major Concerns:

1. Vague Explanation of Assumptions: The manuscript fails to explicitly state the assumptions underlying different methods for detecting epistasis, despite the title being 'Are we making the right assumptions about epistasis?'.

Throughout the manuscript, assumptions are vaguely mentioned as 'assume a specific form' or 'some assumption,' but the exact nature of these assumptions is not specified. This lack of detail makes it difficult for readers to evaluate whether these assumptions are correct. The only clear exception is the discussion on population imbalance, where some methods assume a balanced population, which is well explained.

We thank the reviewer for their pertinent comment. We agree that the title of the manuscript is not a good reflection of the content of the manuscript. We have thus changed the title of the manuscript to “Considerations in the search for epistasis”. We feel that this encompasses the content better and doesn’t lead the reader to expect explicit definitions of assumptions in epistasis throughout the manuscript.

We have also made some edits to add detail regarding assumptions that are being made regarding epistasis in section 1.1:

“Models that identify epistatic interactions from genotype data nearly always make assumptions on the form of the epistatic relationship. There is a gradient in current approaches of epistasis detection; from models assuming a specific form of epistasis (e.g., BOOST[1], BitEpi [2], Fiuncho [3], IRELAND [4,5] to models that learn an epistatic relationship of any form (e.g., [6–9]). These assumptions are described in the paragraphs below. “

“Approaches that assume a specific form of epistatic interaction, for example pairwise, triplet or quadruplet interactions, are often easier to understand and can provide directly interpretable outcomes.”

And:

“On the other hand, freeform approaches such as deep neural networks (DNNs) [19,20,27,28], as supported by mathematical theorems (“the universal approximation theorem, e.g. [29,30], can approximate arbitrary functional relationships, thereby in theory able to not impose any assumption in terms of functional relationships driving epistasis. This makes them more flexible and less prone to computational limits. In practice, however, these approaches require additional steps, perhaps yet to be developed, to not only implicitly capture, but also provide an explicit description of the epistatic interaction [18]. Because DNNs tend to use a large number of input variables in their phenotype prediction, they arguably assume a highly polygenic or even omnigenic trait [29] in practice.”

And:

“These approaches make mild assumptions on the functional form of the epistatic interaction, allowing them to deal with higher-order interactions. For random forests, these assumptions include that SNPs forming interactions must be independent of each other.. Random forests also assume that the relationship between epistasis and genetic variants can be described as a combination of decision trees, while support vector machines assume that one can separate cases from controls by using a hyperplane in the (transformed) variant space. “

2. Scope of Epistasis: The manuscript focuses on genetic interactions in disease heritability, as indicated in Line 57 and Section 2. The hypothesis that "true epistatic interactions will be observable in both model systems and case-control cohorts" (Line 59, Section 2) is only valid within this context. This is because epistasis can vary in different environments and cell types (as cited in ref 77). However, the specific scope of the review is not clearly stated in the title, abstract, or introduction, leading to potential confusion for readers.

We thank the reviewer for this observation and we have thus clarified the scope of the review through changes to the title, abstract and introduction. Specific changes to the introduction are addressed in the next comment. The new title and abstract are copied below.

Title:

“Considerations in the search for epistasis”

Abstract:

“Epistasis refers to changes in the effect of a unit of genetic information (such as a single nucleotide polymorphism or a gene) on phenotype, dependent on the context of other genetic units. Such interactions are both biologically plausible and good candidates to explain observations which are not fully explained by an additive heritability model. However, the search for epistasis has so far largely failed to recover this missing heritability. We identify key challenges and propose that future works need to leverage idealised systems, known biology and even previously identified epistatic interactions, in order to guide the search for new interactions. “

3. Introduction:

The authors discuss epistasis in the context of missing heritability. Since many other factors, besides epistasis, contribute to missing heritability, the authors should also mention genotype-environment interactions to avoid misleading readers who are not experts in genotype-phenotype relationships and heritability.

We thank the Reviewer for this valuable clarification and we have updated this section of the Introduction as follows:

“Missing heritability commonly refers to the gap between measured broad-sense heritability and heritability calculated by adding together the individual contributions of phenotype-associated SNPs genomewide (i.e., narrow-sense heritability). Missing heritability is important because it infers that we have an incomplete understanding of the genetic basis of health and disease. A number of possibilities could explain this missing heritability, including gene-environment interactions. Epistatic interactions another candidate to explain a proportion of missing heritability but an alternative explanation is that current knowledge is simply missing the statistical power to discover all important additive effects. However, there is good observational evidence for epistasis; for example from large-scale screens in yeast studying the effect of combinations of individual gene knockouts [10,11].”

4. Section 1.1 Formula:

The formula presented is problematic. It is described as 'epistasis formulated mathematically,' implying that y represents epistasis. However, y typically symbolizes the quantitative phenotype, and 'e' (or any other symbol) represents epistasis. The term βα(a) is defined as parameters to be estimated, but their biological significance is not explained. It appears that βα(a) signifies epistasis; if so, this needs clarification.

We thank the reviewer for pointing out the need for further clarification. Indeed, y denotes the phenotype, and was intended as such in this formula. Furthermore, (a) denotes the magnitude of the epistatic effect of a set of variants indicated by the vector a.

Intrinsic Relationship Between N and d: The manuscript does not address the theoretical relationship between N(number of SNPs) and d (the highest order interaction). For example, theoretically, the highest-order interaction could be d =N−1. If the authors mean that one can set a maximum highest-order interaction to evaluate (which should be smaller or equal to d=N-1), this should be explicitly stated. Indeed, one can set d such that it places a maximum on the highest order interaction, which can be at most N, where setting d=N reflects an omnigenic model.

Formula (1): In lines 57-60, shouldn't the term β7(x1)^1(x2)^1(x3)^1 also be considered? Otherwise, it appears that only the first-order interaction terms (between two SNPs) are included, without accounting for three-way interactions (involving all three SNPs together).

In the example, d=2, hence the three-way interaction is not included.

In order to address the reviewers comments on the formula in Section 1.1, we have adapted the text as follows:

“Extending the formulation used in [12], a phenotype impacted by epistasis can be formulated mathematically as:

y=a A (a)i {1,...,N}xi ai ,

where N is the total number of SNPs in the data, xi encode the SNP information (e.g. allelic dosage), y symbolizes the phenotype, and

A:={a{0,1}N: 1Tad} with d the order of the highest-order interaction. The parameter d allows one to choose a maximum order for the epistatic interaction, which can be at most N. (a) are the parameters to be estimated denoting the magnitude of the epistatic effect of the variants corresponding to the vector a, where (a) is the index corresponding to the vector a if one were to order all elements of A. The vector a thus indicates which variants are included in the (a)th interaction. For example, in the case of N=3 and d=2 this would give:

y=0 x10x20x30+1 x11x20x30+2 x10x21x30+3 x10x20x31+4 x11x21x30+5 x11x20x31+6 x10x21x31

=0 +1 x1+2 x2+3 x3+4 x1x2+5 x1x3+6 x2x3. (1)

Note that since d=2, the interaction between all three variants is not included.“

5. The manuscript would benefit from additional tables or boxes. Currently, it is unclear what specific assumptions are made by each method of detecting epistasis.

We thank the author for their comment. We agree that a table would nicely summarise the assumptions made by different methods/tools for identifying epistasis. We have summarised this information in Table 1. And added reference to this table in the text below

“Models that identify epistatic interactions from genotype data nearly always make assumptions on the form of the epistatic relationship (Table 1).”

Throughout the manuscript, particularly in the first part, different methods of detecting epistasis are described with phrases like 'assume a specific form' or 'milder assumption', but the exact nature of these forms and assumptions is not specified.

6. Including a table or box summarising this information would provide clarity.

We have addressed this comment with our answer to the previous comment.

7. The first equation (line 41) should be indexed so that the current equation (1) becomes equation (2).

We thank the reviewer for their sharpness and have adjusted the indexing.

8. It is unclear whether the colors of the circles in the lower panels have any significance. If they do, this should be explained.

We apologise for the lack of clarity. The legend to Figure 2 has been updated as follows:

“Figure 2. Example of how generative modelling can be employed to hunt for genetic interactions. Most deep generative models are made of two elements: the Encoder, which reduces dimensionality, and the Decoder, which can generate genetic profiles in silico (top panel). We present three problems where generative models can be employed. Interpretability: The output of the encoder, and input of the decoder, can be interpreted and related to phenotypes of interest. Perturbation: A patient’s genetic profile can be perturbed in silico and passed through the encoder. For instance, a patient with two wild-type alleles (green circles) can be modified by induction of A or B (orange circles) or both at the same time (red circles). Study of the corresponding perturbation in the latent space can help prioritise potentially interacting genetic pairs. Optimization: Finally, a deep generative model could be directly employed inside an optimization strategy geared towards finding epistatic interactions, benefiting from two advantages of deep generative models: the auto-differentiation of the decoder, and the continuous character of the latent space.”

Reviewer #2:

1. In "Are we making the right assumptions about epistasis?", Balvert, Cooper- Knock and colleagues describe a couple of key challenges to detecting epistatic interactions in genomic data. They go on to propose methodological approaches and research infrastructure that would help to identify epistatic interactions in future research. As stated in the introduction, this article summarizes key discussions and conclusions from a recent workshop on this topic, so it will likely be of interest to researchers in the field focusing on the role of epistasis in phenotypic and disease variation in humans.

The authors primarily focus on the role of epistasis in disease heritability estimation and interpretation. While this is certainly an important topic in human medical genetics, it may be worth pointing out that epistasis plays an important role in broader evolutionary theory as well.There are many examples of the role of epistasis in evolutionary biology, both theoretical and empirical, that could be highlighted and potentially broaden the appeal of the review. Many of the challenges for detecting epistatic interactions in non-human-disease settings are the same as those discussed here.

This review article is in large parts well-written, though it could probably use a careful reading for language and clarity. Most sections have a good balance of the "big picture" challenges and suggested approaches to tackling those challenges, and often provide helpful examples or spell out some of the details. However, I believe a few of the sections could be significantly improved (listed below).

We thank the Reviewer for their comments.

2. Section 2.2: While it is pointed out that both intergenic and intragenic epistatic interactions have been documented, the title of the section, "Should we focus on intergenic vs intragenic interaction separately?", (despite being a bit confusingly worded) isn't actually answered. Or rather, the proposed solution is given in a single sentence without justification or detailed expansion. I was left wondering what the point of this section is.

In response to the Reviewer’s comment we have streamlined this section and merged it into the previous section which is entitled: ‘2.1 Should a search for epistasis start with biological observations?’ The difference between intergenic and intragenic epistasis encompasses relatively well understood biology, and therefore we chose to highlight it in the context of this section where we are considering how to use known biology to facilitate the search for epistasis.

3. Section 2.3: A database of epistasis. The authors make an argument for such a database and suggest guidelines and approaches for making it useful to researchers, in addition to challenges. Is such a database in the works? If so, and especially if it is a product of or was first envisioned in the course of this workshop, that could be discussed here.

We thank the reviewers for this comment. We did allude to the need for a comprehensive database of epistasis in our manuscript. Building such a database was beyond the scope of a 5 day workshop, therefore we focused on considerations that should be made to guide the community should they wish to develop such a database in future. We have also added a new citation in section 2.2 for an epistasis database that was published in September 2024, as well as a sentence describing it. The new text is pasted below:

“However, most studies report genome-wide epistasis which are restricted to one organism or phenotype-specific such as Alzheimer’s disease [13,14] or in cancer [15], and are not collated and standardised into a single database. A database that does exist for epistasis across multiple diseases is driven by a single methodological approach (https://epistasis-disease-atlas.com, [16]), therefore is limited in the types of epistatic interactions it contains.”

4. Title: The title doesn't seem to be all that connected to the majority of the content of the review.

We propose a new title: ‘Considerations in the search for epistasis’.

5. Figure 1: I don't find this figure particularly intuitive or informative. I do think that design such conceptual figures is very challenging, but as presented, I don't see it as providing any information beyond a "word cloud". It was difficult to understand the lines connecting the different elements, and the legend didn't clarify things for me (although, such a figure is ideally understandable even without reading the legend).

We apologise for the lack of clarity. The legend to Figure 1 has been updated as follows:

“Figure 1. Key considerations for a comprehensive consideration of epistasis. Epistasis is posed to play a key role in genetic architecture and in the missing heritability problem. In this review, we look at epistasis from two perspectives: driven by genetic information (green circles) and by biological observations (blue circles). We discuss how genetic data can be used in a functional form or in generative models to detect epistasis, and that the inclusion of population structure information derived from genetic data is crucial. On the other hand, the discovery of epistasis can also be informed by biological observations. Ideally, both sides will lead to better detection of epistasis, ultimately leading to a key resource that is a database of epistatic interactions.”

6. I struggled to wrap my head around the interpretability aspect of the latent space from the encoder. It could be helpful to make this a more concrete, either with an example or hypothetical, for those of us less familiar with interpreting machine learning or generative models.

Response: Thank you. We may have not been sufficiently clear on that point. The idea is to not only train the encoder-decoder architectures to appropriately reconstruct the original data from the latent embeddings. Instead, one attaches interpretable labels (such as diseased or not) to the original data, and does not only train the encoder- decoder architecture for accurate reconstruction, but also for accurately distinguishing the embeddings according to their labels in terms of distances in the latent space. This means, for example, that latent embeddings referring to diseased data are close to each other, while being relatively far away from latent embeddings referring to non- diseased data.

This can be achieved by modifying the loss functions the training of the encoder- decoder architecture relies on. Currently popular options are the integration of contrastive loss functions that structure / compartmentalise / cluster the latent space according to phenotypes of interest. Once having mapped new data into latent space, one can interpret the new data according to their location in latent space relative to the spatial areas that refer to the phenotype labels.

Note that such protocols are crucial in the perturbation analyses later on. If modifying variants in non-additive constellations changes assignment to clusters in latent space, epistasis might be in effect. So this suggests to inspect these variants in further analyses.

We have added the following explanation to the manuscript:

“In more concrete detail, using interpretable labels (such as diseased / non-diseased), one trains the encoder to map differently labelled data to distant parts of the latent space, while placing identically labelled data near to each other. Such training procedures can be implemented by the integration of contrastive loss functions [82] into the training of the encoder-decoder architecture. As per their definition, contrastive loss functions are exactly the drivers that keep similar things together, while keeping separate things apart when embedding data into latent space. As a result, the interpretability of the model obtained by an appropriately structured continuously- valued latent space could be instrumental in increasing the power of standard analysis (Figure 2, Interpretability).”

Reviewer #3:

1. Balvert et al present a well written overview of many issues regarding the reality of and the detection of epistasis. The likelihood that epistasis is an important factor in phenotypic variation is a long standing issue especially among human geneticists and as such makes this contribution overall worthy. Below are several comments I think should be more explicitly dealt with in the paper that may clarify some issues.

The first point is that in the abstract it is stated that the role of epistasis in phenotypic variation is controversial. Although true to some extent, this is predominantly true among human geneticists. Research on non-human systems is generally accepting of epistasis.

We thank the Reviewer for their comment and have reworded the abstract to remove this sentence and have added citations and text of epistasis in non-human systems in the introductions:

“However, there is good observational evidence for epistasis; for example from large- scale screens in yeast studying the effect of combinations of individual gene knockouts [10,11].”

2. Another point both in the abstract and introduction is the role of epistasis in the missing heritability. Most arguments about missing heritability have focused on narrow sense heritability, which of course does not explicitly include epistasis. The authors should clarify the role of epistasis in heritability - both, broad where it is explicitly considered and narrow where it may be included, but only implicitly. Specifically, detecting a significant additive model is not necessarily inconsistent with an underlying true epistatic genetic model. The question relates to what is being addressed, understanding genotype-phenotype maps or simply trait prediction.

We thank the Reviewer for this insight and have specified that our argument about missing heritability is the gap between measured broad-sense heritability and narrow- sense heritability. We have also highlighted that the question being addressed is the biological understanding of the genotype-phenotype relationship.

“Broad-sense heritability refers to heritability measured by comparison of concordance rates for phenotype between monozygotic and dizygotic twins who share 100% or 50% of their genetics, respectively [17]. Missing heritability commonly refers to the gap between measured broad-sense heritability and heritability calculated by adding together the individual contributions of phenotype-associated SNPs genomewide (i.e., narrow-sense heritability). Missing heritability is important because it infers that we have an incomplete understanding of the genetic basis of health and disease.”

3. In the discussion of epistasis in yeast a very old citation is used. This is fine but more recent ones, such as, should also be included. For example, Kuzmin et al Science 2018 and Costanzo et al Science 2016 - there are several others from this group alone. This other work also brings up the point of merging detection of epistasis with explicit pathway analyses - not discussed in the paper. This is however key to using biological knowledge to understand epistasis and whether it is important.

We thank the Reviewer for this valuable point. We have added the citations mentioned and we have included a consideration of pathway analysis together with our consideration of intergenic/intragenic epistasis. The new text reads:

“Many double mutant genetic knockout screens have been performed, both in model organisms and human cell lines [18]. The problem with the use of organisms for epistasis detection is, again, the size of the search space. Extensive large-scale screens have been performed in yeast, focusing on large-scale characterisation of cells with combinations of two or more individual gene knockouts or temperature alleles [10,19,20]. Such screens have proved useful, for example in delineating biological pathways containing genes with similar interaction profiles. However, they have not, as yet, provided the scale necessary for an exhaustive search for epistasis. Indeed higher organisms such as mice are not at all suitable for large scale screens due to the practical undertaking involved in exhaustive characterisation. …

An important and well understood biological consideration is the separation between intergenic and intragenic mechanisms of epistasis. There is good evidence for intragenic interactions such as haplotypes associating with altered gene expression depleted for deleterious coding alleles [21]. Similarly, there is evidence for intergenic epistasis, particularly between genes of similar function [22], where an established example concerns mutations within different haemoglobin beta-chains [23]. Intergenic epistasis across different functional pathways also tends to be the result of compensatory adaptation [24] and typically genes within the same pathway have a similar interaction profile [19]. The problem with separating intergenic and intragenic epistasis is that both are combined in real-world biological systems. To circumvent this, we suggest a stepwise approach where intergenic epistasis is analysed and identified before searching for intragenic epistasis within each intergenic interaction.”

4. The issue of population structure and epistasis detection has also been addressed in prior literature in terms of how varying allele frequency of and interacting par affects detection likelihood of detecting marginal effects in spite of an underlying interactive model. For example, CS Greene et al PLOS One 2009 perform simulations to show that background is important for both detection of marginal effects but also interaction. This goes to the point above - does detecting additive effects nullify the likelihood of an underlying interaction. In some ways this is the core of the problem.

We thank the Reviewer for bringing our attention to the citation mentioned. We have included this in the section on population structure (“Furthermore, previous research has shown that a slight change in the allele frequency of a SNP results in a substantial decrease in power to replicate the main effects of said SNP when there is an underlying epistatic model”).

We have also made the point that the presence of additive effects does not necessarily rule out the possibility of an underlying epistatic model in the introduction (“and the presence of an additive model does not necessarily rule out the possibility of an underlying epistatic model”)

5. Lastly, the authors should note in the discussion of animal models and cell lines that these are interesting but not necessarily reflective of nature. In these experiments the backgrounds are usually fixed and in general when examined interactions will be found if there are any kind of biological interactions. However, in outbreeding samples such as case-control studies even if present the same biological interactions may be missed by the resilience of the genetic structure of the population. Similarly, even if two molecular interact, they can only be detected if both are variable in a given sample.

Again the Reviewer makes an excellent point. We have updated this section as follows:

“We assert that conclusive evidence for the role of epistasis in determining disease heritability has come from interactions that have been identified in large case-control cohorts and model systems such as cell lines and organisms. We hypothesise that true epistatic interactions will be observable in both model systems and case-control cohorts. However, false positive epistatic interactions may be more likely in case- control datasets where, for example, population structure is imperfectly matched. On the other hand, the potential challenge with model systems is knowing whether the readout and the cell / tissue context are a correct approximation of disease. Indeed, a model system may suggest an epistatic interaction which is specific to the genetic background of the model organism and may not be important in an outbred population.”

6. Overall, this is an excellent overview but not comprehensive. Although being completely comprehensive is not possible in such a short piece, several ideas as noted above should probably be addressed to improve the completeness of the arguments.

We thank the Reviewer for their helpful comments. We think that our work has been improved by incorporating the Reviewer’s suggestions.

1. Wan X, Yang C, Yang Q, Xue H, Fan X, Tang NLS, et al. BOOST: A fast approach to detecting gene-gene interactions in genome-wide case-control studies. Am J Hum Genet. 2010;87:325–40.

2. Bayat A, Hosking B, Jain Y, Hosking C, Kodikara M, Reti D, et al. Fast and accurate exhaustive higher-order epistasis search with BitEpi. Sci Rep. 2021;11:1–12.

3. Ponte-Fernández C, González-Domínguez J, Martín MJ. Fiuncho: a program for any-order epistasis detection in CPU clusters. J Supercomput. 2022;78:15338–57.

4. Balvert M. Iterative Rule Extension for Logic Analysis of Data: An MILP-Based Heuristic to Derive Interpretable Binary Classifiers from Large Data Sets. INFORMS J Comput [Internet]. 2024; Available from: https://doi.org/10.1287/ijoc.2021.0284

5. Pattin KA, White BC, Barney N, Gui J, Nelson HH, Kelsey KT, et al. A computationally efficient hypothesis testing method for epistasis analysis using multifactor dimensionality reduction. Genet Epidemiol. 2009;33:87–94.

6. Poelwijk FJ, Krishna V, Ranganathan R. The Context-Dependence of Mutations: A Linkage of Formalisms. PLoS Comput Biol. 2016;12:e1004771.

7. Aghazadeh A, Nisonoff H, Ocal O, Brookes DH, Huang Y, Koyluoglu OO, et al. Epistatic Net allows the sparse spectral regularization of deep neural networks for inferring fitness functions. Nat Commun. 2021;12:5225.

8. Motsinger-Reif AA, Fanelli TJ, Davis AC, Ritchie MD. Power of grammatical evolution neural networks to detect gene-gene interactions in the presence of error. BMC Res Notes. 2008;1:65.

9. Li X, Liu L, Zhou J, Wang C. Heterogeneity Analysis and Diagnosis of Complex Diseases Based on Deep Learning Method. Sci Rep. 2018;8:6155.

10. Segrè D, Deluna A, Church GM, Kishony R. Modular epistasis in yeast metabolism. Nat Genet. 2005;37:77–83.

11. Sameith K, Amini S, Groot Koerkamp MJA, van Leenen D, Brok M, Brabers N, et al. A high-resolution gene expression atlas of epistasis between gene-specific transcription factors exposes potential mechanisms for genetic interactions. BMC Biol. 2015;13:112.

12. Cordell HJ. Epistasis: what it means, what it doesn’t mean, and statistical methods to detect it in humans. Hum Mol Genet. 2002;11:2463–8.

13. Lundberg M, Sng LMF, Szul P, Dunne R, Bayat A, Burnham SC, et al. Novel Alzheimer’s disease genes and epistasis identified using machine learning GWAS platform. Sci Rep. 2023;13:17662.

14. Wang H, Bennett DA, De Jager PL, Zhang Q-Y, Zhang H-Y. Genome-wide epistasis analysis for Alzheimer’s disease and implications for genetic risk prediction. Alzheimers Res Ther. 2021;13:55.

15. Park S, Lehner B. Cancer type-dependent genetic interactions between cancer driver alterations indicate plasticity of epistasis across cell types. Mol Syst Biol. 2015;11:824.

16. Hoffmann M, Poschenrieder JM, Incudini M, Baier S, Fritz A, Maier A, et al. Network medicine-based epistasis detection in complex diseases: ready for quantum computing. Nucleic Acids Res. 2024;52:10144–60.

17. Verweij KJH, Yang J, Lahti J, Veijola J, Hintsanen M, Pulkki-Råback L, et al. Maintenance of genetic variation in human personality: testing evolutionary models by estimating heritability due to common causal variants and investigating the effect of distant inbreeding. Evolution. 2012;66:3238–51.

18. Mackay TFC. Epistasis and quantitative traits: using model organisms to study gene-gene interactions. Nat Rev Genet. 2014;15:22–33.

19. Kuzmin E, VanderSluis B, Wang W, Tan G, Deshpande R, Chen Y, et al. Systematic analysis of complex genetic interactions. Science [Internet]. 2018;360. Available from: http://dx.doi.org/10.1126/science.aao1729

20. Costanzo M, VanderSluis B, Koch EN, Baryshnikova A, Pons C, Tan G, et al. A global genetic interaction network maps a wiring diagram of cellular function. Science. 2016;353:aaf1420–aaf1420.

21. Cisneros AF, Gagnon-Arsenault I, Dubé AK, Després PC, Kumar P, Lafontaine K, et al. Epistasis between promoter activity and coding mutations shapes gene evolvability. Sci Adv. 2023;9:eadd9109.

22. Mapping the genetic landscape of human cells. Available from: https://www.cell.com/cell/pdf/S0092-8674(18)30735-9.pdf

23. Tufts DM, Natarajan C, Revsbech IG, Projecto-Garcia J, Hoffmann FG, Weber RE, et al. Epistasis constrains mutational pathways of hemoglobin adaptation in high- altitude pikas. Mol Biol Evol. 2015;32:287–98.

24. Rojas Echenique JI, Kryazhimskiy S, Nguyen Ba AN, Desai MM. Modular epistasis and the compensatory evolution of gene deletion mutants. PLoS Genet. 2019;15:e1007958.
